# The Epidemiological Characteristics and Dynamic Transmission of Dengue in China, 2013

**DOI:** 10.1371/journal.pntd.0005095

**Published:** 2016-11-07

**Authors:** Shaowei Sang, Shasha Wang, Liang lu, Peng Bi, Ming Lv, Qiyong Liu

**Affiliations:** 1 Clinical Epidemiology Unit, Qilu Hospital of Shandong University, Jinan, People’s Republic of China; 2 Institute of Public Health and Management, Weifang Medical University, Weifang, People’s Republic of China; 3 State Key Laboratory of Infectious Disease Prevention and Control, Collaborative Innovation Center for Diagnosis and Treatment of Infectious Diseases, National Institute for Communicable Disease Control and Prevention, Chinese Center for Disease Control and Prevention, Changping, Beijing, People’s Republic of China; 4 School of Public Health, Anhui Medical University of China, People’s Republic of China; 5 School of Public Health, The University of Adelaide, Adelaide, Australia; University of California, Berkeley, UNITED STATES

## Abstract

**Background:**

There was a dengue epidemic in several regions of China in 2013. No study has explored the dynamics of dengue transmission between different geographical locations with dengue outbreaks in China. The purpose of the study is to analyze the epidemiological characteristics and to explore the dynamic transmission of dengue in China, 2013.

**Methodology and Principal Findings:**

Records of dengue cases of 2013 were obtained from the China Notifiable Disease Surveillance System. Full E-gene sequences of dengue virus detected from the outbreak regions of China were download from GenBank. Geographical Information System and heatmaps were used to describe the epidemiological characteristics. Maximum Likelihood phylogenetic and Bayesian phylogeographic analyses were conducted to explore the dengue dynamic transmission. Yunnan Province and Guangdong Province had the highest imported cases in the 2013 epidemic. In the locations with local dengue transmission, most of imported cases occurred from June to November 2013 while local dengue cases developed from July to December, 2013. There were significant variations for the incidences of dengue, in terms of age distributions, among different geographic locations. However, gender differences were identified in Guangzhou, Foshan and Xishuangbanna. DENV 1–3 were detected in all locations with the disease outbreaks. Some genotypes were detected in more than one locations and more than one genotypes have been detected in several locations. The dengue viruses introduced to outbreak areas were predominantly from Southeast Asia. In Guangdong Province, the phylogeographical results indicated that dengue viruses of DENV 1 were transmitted to neighboring cities Foshan and Zhongshan from Guangzhou city, and then transmitted to Jiangmen city. The virus in DENV 3 was introduced to Guangzhou city, Guangdong Province from Xishuangbanna prefecture, Yunnan Province.

**Conclusions:**

Repeated dengue virus introductions from Southeast Asia and subsequent domestic dengue transmission within different regions may have contributed to the dengue epidemics in China, 2013.

## Author Summary

Dengue is the most prevalent and rapidly spreading mosquito-borne viral disease. As an imported disease in China, the imported cases play a vital role for the local dengue transmission. There were dengue outbreaks in three Provinces (covering nine Cities/Prefectures) of China in 2013, with several regions had their first dengue outbreak in history including the one from central China. There has been no study so far to explore the dengue transmission dynamics between different regions in China. The purpose of the study is to describe the 2013 dengue epidemiological characteristics and to explore the transmission dynamics of dengue viruses between epidemic focus. The study results indicated that repeated dengue virus introductions from Southeast Asia and subsequent domestic dengue transmission within different regions may have contributed to the dengue epidemics in China, 2013. Population movement could have played a critical role in dengue dynamic transmission, which introduced dengue viruses to non-epidemic areas at broad or finer spatial scales. Therefore, it should be considered in the design of mosquito eradication campaign for dengue control and prevention.

## Introduction

Dengue is a mosquito-borne viral infectious disease caused by the four antigently distinct serotypes (DENV 1–4), which are mainly transmitted by *Aedes aegypti* and *Aedes albopicuts*. Dengue is endemic in more than 100 countries in tropical and subtropical areas, especially in Southeast Asia, the Americas, the Western Pacific, Africa and Eastern Mediterranean regions [1]. Because of unprecedented population growth, uncontrolled urbanization, spread of the mosquito vectors and the population movement, the incidence of dengue has increased dramatically in the past 50 years [2]. It is estimated that 390 (95% CI: 284–528) million people have dengue virus infections with 96 (95% CI: 67–136) million cases annually worldwide [3].

The dynamics of dengue transmission depends on the interactions among hosts, viruses, vectors and environmental factors. Given the restricted range of mosquito flying distance [4, 5], population movement may play a critical role for dengue transmission. At broad spatial scales (e.g., national, international), human movements may make dengue virus being introduced and reintroduced into a region with lower herd immunity [6]. Travel acquired cases were repeatedly imported to Europe from Africa, Southeast Asia and the Americas [7–9]. Local dengue transmission has occurred in Europe for the first time in many decades, with indigenous cases reported in France and Croatia in 2010 [10, 11]. In addition to sporadic cases, dengue outbreak also occurred in Europe. For example, an outbreak with more than 2,000 cases happened in Madeira, Portugal in 2012, which was most probable origin of Venezuela [12]. Aware of the importance of air travel in dengue transmission, researchers developed simple models to estimate the importing risk of dengue or even the possible origin of importation in Europe [13, 14]. In Asia, because of the reintroductions of dengue viruses from Southern Vietnam where dengue is endemic, Northern Vietnam had dengue epidemics occurred frequently [15]. At finer spatial scales (regional, intra-urban, neighborhood), population movements associated with work and recreation are important for dengue transmission [6], and house-to-house human movements may shape spatial patterns of dengue incidence, causing significant heterogeneity in dengue incidence [16].

There was no dengue case notified from 1949 to 1977 in China until an outbreak occurred in Guangdong Province in 1978. Since then, dengue has been detected for nearly forty years in China. It was prevalent in Southern China including Guangdong Province, Hainan Province and Guangxi Province in 1980s. Since 1990, dengue was predominantly occurred in Guangdong Province. Geographically, the dengue outbreaks have expanded gradually from Guangdong, Hainan and Guangxi in Southern coastal regions of China to the relatively Northern regions including Fujian, Zhejiang Provinces and to the relatively Western region Yunnan Province [17]. Neighboring to Myanmar, Laos and Vietnam, Yunnan Province had its first dengue outbreak with 56 cases reported in 2008, of which most were imported cases from Myanmar [17]. In 2013, Guangdong, Yunnan and Henan Provinces had dengue outbreaks. This was the first dengue outbreak in Henan Province, the most northern Province with dengue local transmission. The 2013 outbreak in Yunnan was the second outbreak and also the first severe dengue outbreak in the Province. Guangdong Province has the highest dengue incidence with cases reported every year since 1997, with the most prevalent in 2013.

Our previous study has proven that dengue was still an imported disease in China [18], and the epidemics were probable to be originated from overseas. Three individual studies have reported the dengue outbreaks occurred in Yunnan and Henan Provinces in 2013 [19–21]. However, no study tried to explore the transmission dynamics between locations. The purpose of the study is to describe the 2013 epidemiological characteristics and to explore the possible origins of the epidemics, and the dynamics of dengue viruses between epidemic focus, which are important to dengue control and prevention.

## Methods

### Ethics statement

Ethical approval for the study was obtained from the Chinese Center for Control and Prevention Ethical Committee (No.201214) and patient data in the study were de-identified and analyzed in aggregated format.

### Data collection

Records of notified dengue cases of 2013 were obtained from the China Notifiable Disease Surveillance System, including age, gender, occupation, date of onset, type of diagnosis, local case or not. At the study areas, a dengue case is defined as an imported case for which the patient had traveling history to a dengue affected area and reported being bitten by mosquitoes within 15 days of the onset of illness. In some cases, importation is defined based on laboratory results showing that the infecting dengue virus had a high sequence similarity in the preM/E region compared with viruses isolated from the putative source region where the patient had traveled to. Otherwise, the dengue case is considered to be a local case [22].

Henan, Yunnan and Guangdong Provinces had dengue outbreaks in 2013. Full E-gene sequences of dengue virus detected in these Provinces in 2013 were downloaded from GenBank (As of August 25^th^ 2015) (S1 Table). The sequences detected in China were compared with published sequences by using the nucleotide blast program in the NCBI. S2–S4 Tables were the references downloaded with the accession number, collection date and geographical region.

The population data were from the Sixth National Population Census of China conducted by the National Bureau of Statistics of the People’s Republic of China in 2010. The information of epidemiological investigation was downloaded from China Public Health Emergency Management Information System.

### Data analysis

#### Epidemiological analysis

ArcGIS 10.1 (ESRI, Redlands, CA, USA) was used to describe and plot the epidemiological characteristics in the spatial dimension. The imported cases were aggregated at Province level. Incidence of local cases per 100,000 was calculated at city level. Heatmaps were created to plot the time series of imported and local dengue cases, respectively, by R 3.1.1 [23]. The differences of local dengue incidence in age distribution and sex distribution were analyzed by chi-square test.

#### Genotyping

All the sequences were aligned using MAFFT [24]. Maximum Likelihood (ML) trees of each serotype were constructed using PhyML [25], and the best-fit model of nucleotide substitutions was selected on the basis of Akaike Information Criterion (AIC) conducted by jModelTest [26]. The reliability of branching pattern was tested through 1,000 bootstrap sampling.

#### Analysis of spatio-temporal dispersion pattern

The spatial diffusion of each genotype was jointly estimated using the Bayesian Markov chain Monte Carlo (MCMC) statistical framework implemented in the BEAST v1.8.2 package [27]. The phylogeographic diffusion process between state variables was identified using the Bayesian stochastic variable search selection (BSSVS). Analyses were carried out with a constant size tree prior [18], under strict or relaxed (uncorrelated log-normal) clock model. The nucleotide substitution model for every genotype was selected by jModelTest [26]. The MCMC analysis was run for 30 million generations and the convergence of parameters (ESS>200) was assessed with TRACER v1.5 program (http://tree.bio.ed.ac.uk/software/tracer/). Uncertainty in parameter estimates was reflected in the 95% highest probability density (HPD) intervals. The programs TreeAnnotator v1.8.2 and Figtree (http://tree.bio.ed.ac.uk/software/figtree/) were used to summarize the posterior tree distribution and to visualize the annotated maximum clade credibility (MCC) tree, respectively.

## Results

### Epidemiological characteristics of dengue cases in China, 2013

**Fig 1:**
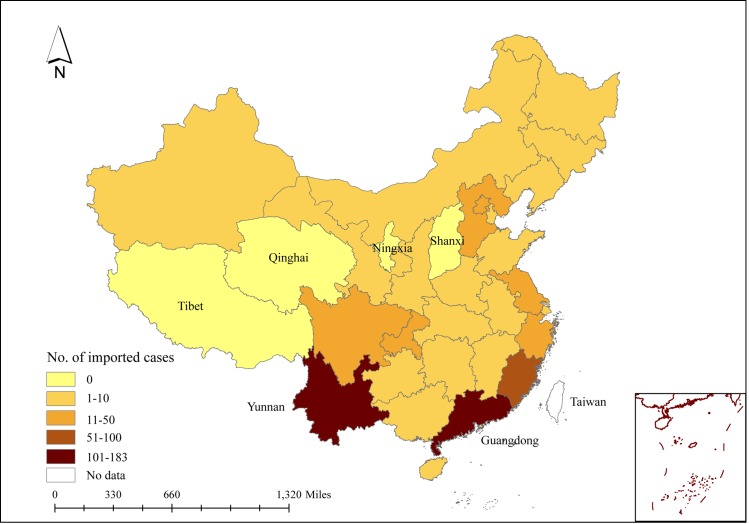
The number of imported cases in China, 2013. Taiwan: data not available.

**Fig 2:**
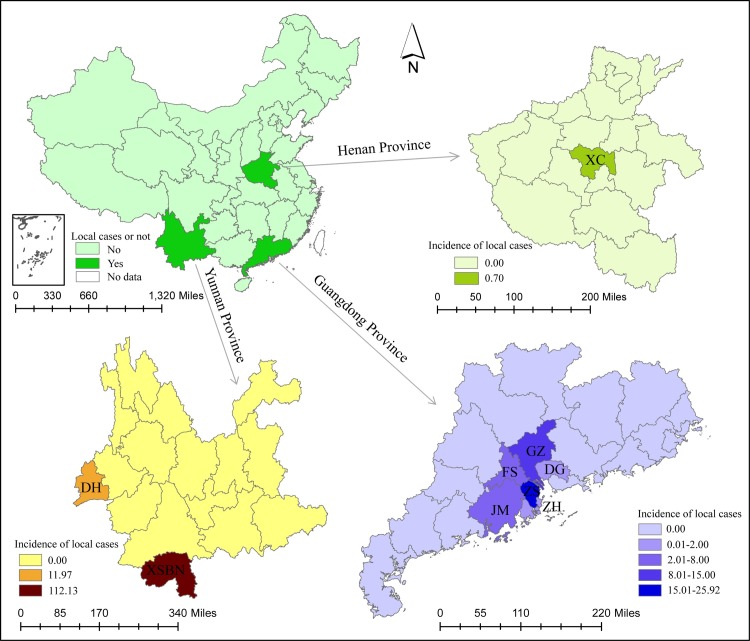
The incidence of local cases in outbreak locations (Incidence/100,000). XC: Xuchang; DH: Dehong; XSBN: Xishuangbanna; GZ: Guangzhou; FS: Foshan; DG: Dongguan; ZS: Zhongshan; JM: Jiangmen; ZH: Zhuhai.

There were 4,779 dengue cases reported in the China Notifiable Disease Surveillance System in 2013, including 543 imported cases and 4,236 local cases. No dengue case was notified in Tibet, Qinghai, Ningxia and Shanxi Provinces. Imported dengue cases were reported in other Provinces with the highest numbers in Yunnan and Guangdong Provinces (Fig 1). In Henan Province, outbreak occurred in central China, Xuchang city with the incidence of 0.70 per 100,000. In Southwestern China, dengue outbreaks occurred in Dehong prefecture and Xishuangbanna prefecture locating in west and south of Yunnan Province with incidence of local cases 11.97 and 112.13 per 100,000, respectively. Dengue outbreaks occurred in Central and South of Guangdong Province (Southern China) including Guangzhou city, Foshan city, Dongguan city, Zhongshan city, Zhuhai city and Jiangmen city. The incidence ranged from 0.12 to 25.92 per 100,000, with Zhongshan city the highest and Guangzhou the second highest incidence (Fig 2).

In the regions with local dengue transmission, imported dengue cases occurred almost all year around, with the most cases happening from June to November—accounting for 93.89% of cases (215/229) (the number of imported cases in the regions with local dengue transmission was 229). Dehong and Xishuangbanna had the most imported cases, accounting for 68.12% (156/229) (S1 Fig). Local dengue cases occurred in July to December. The first local dengue case occurred in Zhongshan city, Guangdong Province (S2A Fig). Gradually, the cities around Zhongshan city all had dengue outbreaks. Meanwhile, dengue outbreaks also hit Xuchang city, Henan Province and Dehong prefecture and Xishuangbanna prefecture, Yunnan Province (S2B Fig).

**Fig 3:**
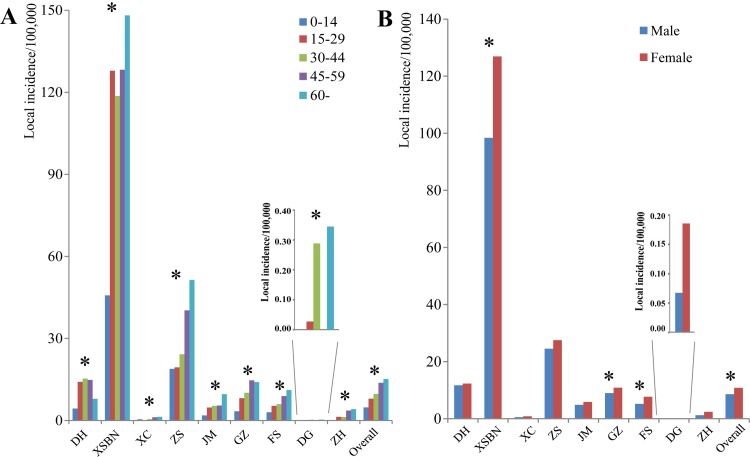
The demographic characteristics of local dengue cases in outbreak regions, China, 2013. A: The age distribution of local incidence; The legend was the age groups. Asterisk meant local incidence had significant difference among different age groups in respective outbreak region. B: The sex distribution of local incidence. Asterisk meant local incidence had significant difference between sex in respective outbreak region. XC: Xuchang; DH: Dehong; XSBN: Xishuangbanna; GZ: Guangzhou; FS: Foshan; DG: Dongguan; ZS: Zhongshan; JM: Jiangmen; ZH: Zhuhai.

The local incidence among different age groups showed significant differences in each outbreak area and the overall incidence in different age groups had a gradual increase with the age (Fig 3A). The local incidence between male and female was significantly different in Xishuangbanna, Guangzhou and Foshan, and the overall local incidence between male and female had significant difference, with more cases in females than in males (Fig 3B).

### Phylogenetic and phylogeographical analysis of genotypes detected in China, 2013

**Fig 4:**
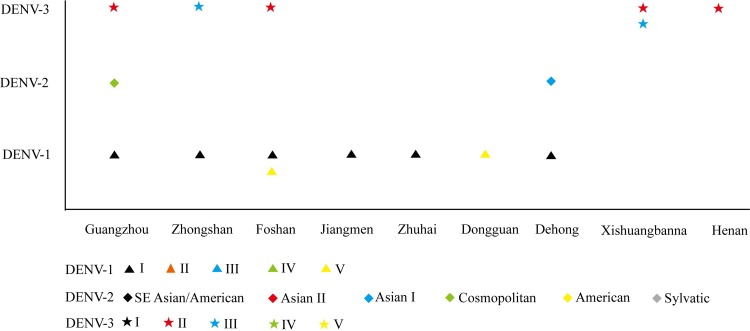
The genotypes of each serotypes detected in dengue outbreak locations, China, 2013. The horizontal axis represented the outbreak locations and the vertical axis represented the genotypes in each serotypes. The shapes represented different serotypes and the colors in each serotype represented different genotypes.

DENV 1–3 were detected in all regions with dengue outbreaks in 2013 (S3–S5 Figs). Among these, DENV 1-I, DENV 1-V, DENV 3-II and DENV 3-III were detected in more than one outbreak regions. More than one genotypes have been detected in several locations (Fig 4).

**Fig 5:**
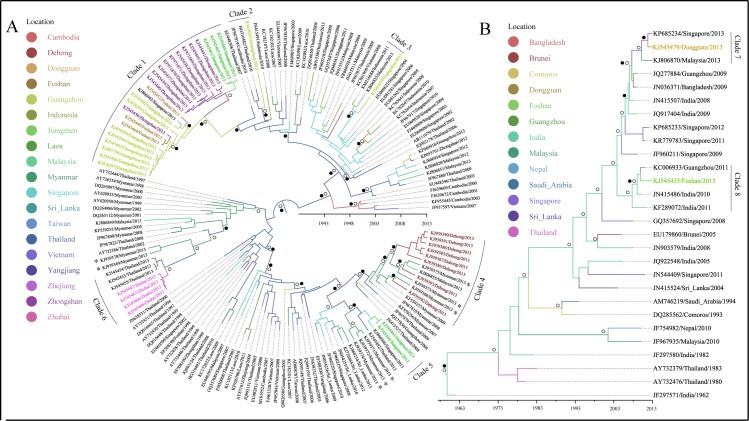
Maximum clade credibility (MCC) trees summarized for DENV 1. A, B represented the MCC trees of genotype I and genotype V, respectively. The colors of the branches corresponded to their probable geographic location (see the legend). Labs with colors represented strains detected in outbreak areas in 2013. Circles indicated posterior probability support ≥ 0.8. Black dots indicate ancestral location state probability ≥ 0.8. Asterisk represented the strains introduced from overseas.

**Fig 6:**
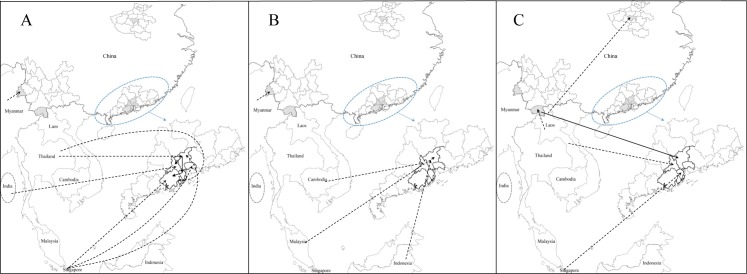
The probable sources of dengue virus detected in China. A, B, C represented the dynamic transmission of DENV 1, DENV 2, DENV 3, respectively. The dashed arrow represented the dynamic transmission of dengue viruses from overseas. The solid arrow represented the dynamic transmission of dengue virus in China.

**Fig 7:**
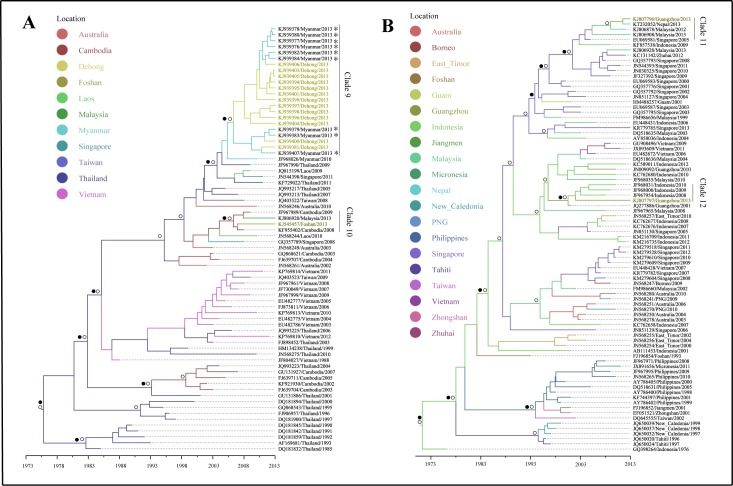
Maximum clade credibility (MCC) trees summarized for DENV 2. A, B represented the MCC trees of Asian I genotype and Cosmopolitan genotype, respectively. The colors of the branches corresponded to their probable geographic location (see the legend). Labs with colors represented strains detected in outbreak areas in 2013. Circles indicated posterior probability support ≥ 0.8. Black dots indicate ancestral location state probability ≥ 0.8. Asterisk represented the strains introduced from overseas. PNG: Papua New Guinea.

**Fig 8:**
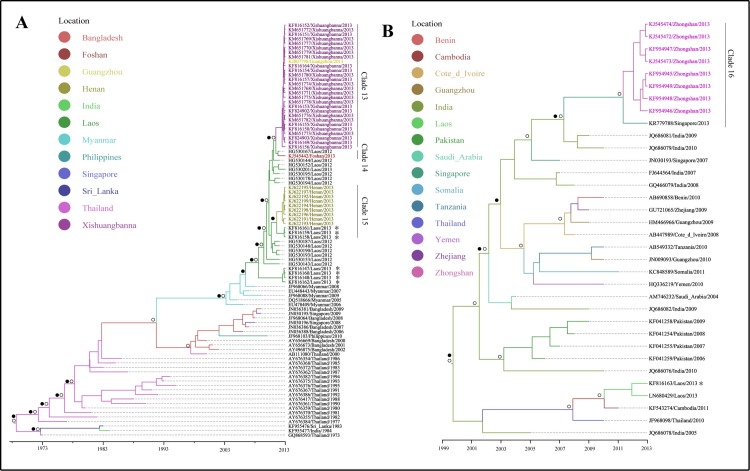
Maximum clade credibility (MCC) trees summarized for DENV 3. A, B represented the MCC trees of genotype II and genotype III, respectively. The colors of the branches corresponded to their probable geographic location (see the legend). Labs with colors represented strains detected in outbreak areas in 2013. Circles indicated posterior probability support ≥ 0.8. Black dots indicate ancestral location state probability 0.8. Asterisk represented the strains introduced from overseas.

The dengue viruses detected in Guangdong Province in Clade 1 showed that Thailand strains were introduced to Guangzhou first, and then transmitted into its neighboring cities: Foshan and Zhongshan, and were then transmitted to Jiangmen from Zhongshan (Figs 5A and 6A). The phylogeographical results indicated that dengue viruses detected in Guangzhou, located in Clade 2, 3, 11, 12, were probably imported from Thailand, Singapore, Malaysia, Indonesia (Figs 5A, 7B, 6A and 6B), while Clade 13 showed that some Guangzhou strain was transmitted from Xishuangbanna, Yunnan Province (Figs 8A and 6C). Dengue viruses detected in Dehong, Yunnan Province (Clade 4, 9) were probably introduced from its neighbor, Myanmar (Figs 5A, 7A, 6A and 6B). Some strains detected in Jiangmen, the strain in Dongguan and some strains in Zhongshan, Guangdong Province, were probably imported from Singapore (Clade 5, 7, 16; Figs 5A, 5B, 8B, 6A and 6C). The outbreak occurred in Zhuhai, Guangdong Province was probably caused by introduced viruses from Thailand (Clade 6, Figs 5A and 6A). The results indicated that some strains detected in Foshan, Guangdong Province were introduced from India, Cambodia and Laos (Clade 8, 10, 14; Figs 5B, 7A, 8A and 6). Fig 8A indicated that the dengue viruses in Xishuangbanna were probably introduced from Laos (Clade 13; Fig 6C). In addition, the viruses detected in Henan Province were also probably imported from Laos (Clade 15; Figs 8A and 6C)

## Discussion

Dengue has been detected in China for nearly 40 years, and has become more serious in these years with increased incidence and expanded outbreak regions. Our previous study has proven that dengue is still an imported disease in China [18], and the results from this study suggested that the introduced dengue cases from overseas may have contributed to the dengue outbreaks. The 2013 outbreaks occurred in different locations of China were mainly due to the introduced viruses from Southeast Asia and domestic dengue transmission within different regions of China.

The demographic characteristics of dengue cases showed that dengue mainly affects adults in China, which were different from that in dengue endemic countries, where adult acquired lifelong immunity [28]. Adults were vulnerable in China given they have more opportunities to expose to mosquitoes when there is lower herd immunity. The sex distribution of dengue varied in different countries, and even in different locations or different epidemic time periods within a country [29–32]. The sex distribution of dengue may be attributed to the relevant demand on the health services of different sexes.

Located in Central China, Henan Province has a temperate climate. This was the first dengue outbreak in the Province. Geographically, it was the northernmost place with dengue outbreak in China, which is almost on the same latitude as Madeira, Portugal where dengue outbreak occurred in Europe. Generally, dengue occurs mainly in tropical and subtropical areas, where suitable climatic conditions play great roles in dengue transmission. Liu-Helmersson found that dengue epidemic potential in temperate was increased with increasing diurnal temperature range [33]. In addition, human movement plays an important role in dengue outbreak in temperate climate region. The dengue outbreak in Henan occurred in a small village with six porcelain factories. The epidemiological investigation showed that all dengue cases had neither travel history, nor collective activities. However, five people from the village worked in Laos where dengue was endemic. These five people returned to their home village in Henan for their vocational leave in May 2013. Although none of them were reported having dengue, two family members of these five people reporting dengue infections, indicating that they may have picked up the virus from the Laos and transmitted to their family members. In addition, the products of porcelain factories were usually sold to Guangzhou and Yunnan Province where dengue was prevalent in 2013 [21]. While phylogenetic and phylogeograhical analyses showed that the Henan strains and Yunnan strains (Xishuangbanna) were separated into different clades with high posterior probability support (Clade 13, 15; Fig 8A). Dengue was epidemic in Laos in 2012–2013 and the epidemic was severe in 2013, which might be attributed to the serotype switch from DENV 1 to DENV 3, and genotype II of DENV 3 was the predominant genotype [34, 35]. The strains detected in Henan Province were genotype II of DENV 3 (Clade 15) and the phylogeographical analysis indicated that the dengue viruses were probably introduced from the Laos with high ancestral location state probability.

Xishuangbanna prefecture, Yunnan Province, neighboring to Myanmar and Laos, had its first dengue outbreak in 2013. The phylogenetic results showed genotype II of DENV 3 caused the outbreak (Clade 13). As mentioned before, the same genotype was prevalent in Laos in 2012–2013. The epidemiological field investigation showed that there were imported cases from Laos and Myanmar at the beginning of the epidemic, and some businessmen has travel history to Laos. The phylogeographical analysis indicated that the viruses detected in Xishuangbanna were probably introduced from Laos. Therefore, because of the repeated introductions and lack of local herd immunity, the viruses imported from Laos probably contributed to the dengue outbreak in Xishuangbanna, Yunnan Province in 2013.

Dehong prefecture, Yunnan Province, bordering Myanmar also had a large dengue outbreak in 2013. The epidemiological analysis showed that there were 245 dengue cases in Dehong, of which 101 cases were imported cases from Myanmar. Most local dengue cases engaged in trade activities with Burmese around. The strains detected in Dehong were from local dengue cases and imported cases, and the imported cases were all from Myanmar [20]. The phylogenetic analysis indicated that the dengue viruses detected in Dehong were classified into genotype I in DENV 1 and Asian I genotype in DENV 2. The ML tree and MCC tree suggested the local cases and some imported cases from Myanmar were clustered together and the phylogeographical analysis indicated that the dengue outbreak occurred in Dehong were caused by two different genotypes, which were both introduced from Myanmar. Therefore, two genotypes co-circulated in Dehong invading from neighboring Myanmar and contributed to the dengue transmission.

Dengue epidemics occurred in Dehong and Xishuangbanna, Yunnan Province were attributed to the dengue viruses transmission across the border, and the main dengue transmission vector *Aede aegypti* existed in these two areas played a critical role in local dengue transmission [36].

In Guangdong Province, local dengue cases occurred in Pearl River Delta of Guangdong (PRD) in 2013, where is one of the most densely urbanized regions in the world [37]. The area is one of the homelands of overseas Chinese, especially to the those live in Southeast Asia, which brings about many travels from there each year. Dengue is endemic in Southeast Asia, with severe dengue outbreaks in Indonesia, Thailand, Singapore and Malaysia in 2013 [38–40]. These countries are tourism resorts and there have been intensive trade activities between these countries and the PRD. The dengue viruses may have imported to PRD frequently from these endemic areas because of the frequent population movement and probably contributed to the dengue outbreaks in these PRD areas. The phylogenetic and phylogeographical results indicated that the strains detected in the PRD areas, especially in Guangzhou showed diversity, and most strains were probably introduced from Singapore, Thailand, Malaysia, Indonesia, Cambodia and Laos (Fig 6).

Neighboring to Guangzhou, Dongguan is characterized by its fast urbanization and manufacturers in southern China. Although dengue infections have been reported in Guangzhou almost every year with four serotypes being isolated, Dongguan was free of dengue until its first dengue outbreak in 2010, which might be caused by viruses imported from Malaysia [41]. For the second dengue outbreak in Dongguan in 2013, although the Dongguan strain, Singapore and Malaysia strain were clustered together, the phylogeographical result indicated that Singapore strain caused the dengue outbreak with high location probability.

Because of the multiple introductions, dengue outbreaks occurred in epidemic seasons in Southern China in the context of suitable weather conditions [42]. Population movement at finer spatial scales contributed to the epidemic foci expansion, and may contribute dengue transmission from one epidemic city to another one within China [6]. The phylogenetic analyses indicated that the dengue epidemic transmitted to neighboring Foshan city southwestward and Zhongshan city southward from Guangzhou city, and then to Jiangmen city from Zhongshan city (Fig 6A). Given the increased economic link and population movement, there existed the probability dengue virus dispersion to Foshan from Guangzhou, because the outbreak in Guangzhou was earlier than that in Foshan. The phylogenetic analysis showed that the outbreak in Zhongshan was caused by two serotypes, with epidemic two peaks. The cases in Zhongshan were located on the border with Guangzhou and the dengue outbreak in Zhongshan was earlier than that in Guangzhou. Therefore, there existed the probability that the first peak occurring earlier than that in Guangzhou was caused by introduced viruses from Singapore (Fig 8B; Clade 16) and the second was probably attributed to the viruses transmitted from Guangzhou. The cases in Jiangmen city mainly occurred in the districts adjacent to Zhongshan city—this could be because that some of the residents working in Zhongshan while living in Jiangmen due to cheaper property price in Jiangmen and higher salary in Zhongshan, which made the dengue viruses transmitted to Jiangmen from Zhongshan feasible.

The phylogeographical analyses showed that Guangzhou not only transmitted viruses to adjacent areas, but also received from other area nationally. The result showed that there existed the probability that dengue virus transmitted to Guangzhou from Xishuangbanna, Yunnan Province. The ML tree and MCC tree both showed the Guangzhou strain was embraced in Xishuangbanna clade. Being the centre for industry, finance, transportation and trade in southern China, there are many migrant workers in Guangzhou, nationally and internationally. Xishuangbanna is a tourism resort, which attracted many people including these from Guangzhou. Given dengue outbreak occurred earlier in Xishaungbanna than that in Guangzhou, the dengue virus in Xishuangbanna probably contributed to dengue transmission in Guangzhou.

This is the first study exploring the dengue dynamics in China, which is critical to understand dengue transmission and will be helpful to prevent and control dengue occurrence in China. Because the E-gene data were downloaded from GenBank, we are unable to obtain the exact epidemiological information of each single sequence. Among the three Provinces with dengue outbreaks in 2013, we tried to assess whether the cases in Yunnan Province were local cases or not from published papers regarding its outbreak [19, 20]. For Henan and Guangdong Provinces, we contacted the Provincial CDCs, and confirmed that the sequences deposited to GenBank were all from local cases. However, we are not able to get the exact onset time for each case.

Dengue is still an imported disease in China. Because of population movement and the close connections between Southern China and Southeast Asia, Southeast Asia is still the main sources of dengue viruses introduced to China. At the background of climate change and the existence of dengue vector, dengue epidemic is not restricted in Southern China any more. The Pearl River Delta of Guangdong are merging more and more close in recent years,which makes dengue virus disperse easily. Therefore, population movement plays a critical role in dengue dynamic transmission, which introduces dengue viruses to non-epidemic areas at broad or finer spatial scales. Apparently relevant dengue control and prevention strategies should be updated.

## Supporting Information

S1 Fig The heatmap of imported cases from locations with local dengue transmission. The bar on right side represented the number of dengue cases.
(TIF)

S2 Fig The heatmap of local cases and the relative time of local transmission in every location. A: Time series of daily local dengue cases. The bar on right side represented the number of dengue cases; B: The plot of relative time of local transmission in every location.
(TIF)

S3 Fig Maximum Likelihood (ML) tree of DENV 1 based on complete E gene nucleotide sequences. The tree is mid-point rooted. Labs with colors represented strains detected in outbreak areas in China, 2013. Asterisk represented the strains introduced from overseas.
(TIF)

S4 Fig Maximum Likelihood (ML) tree of DENV 2 based on complete E gene nucleotide sequences. The tree is mid-point rooted. Labs with colors represented strains detected in outbreak areas in China, 2013. Asterisk represented the strains introduced from overseas.
(TIF)

S5 Fig Maximum Likelihood (ML) tree of DENV 3based on complete E gene nucleotide sequences. The tree is mid-point rooted. Labs with colors represented strains detected in outbreak areas in China, 2013. Asterisk represented the strains introduced from overseas.
(TIF)

S1 Table Strains detected in outbreak areas in China, 2013 (XLSX)

S2 Table DENV-1 references. (XLSX)

S3 Table DENV-2 references. (XLSX)

S4 Table DENV-3 references. (XLSX)
