# Supplementary figures and images for: The Epidemiological Characteristics and Dynamic Transmission of Dengue in China, 2013
Source: PLoS Negl Trop Dis. 2016 Nov 7;10(11):e0005095. doi: 10.1371/journal.pntd.0005095 (PMC5098828; doi:10.1371/journal.pntd.0005095)

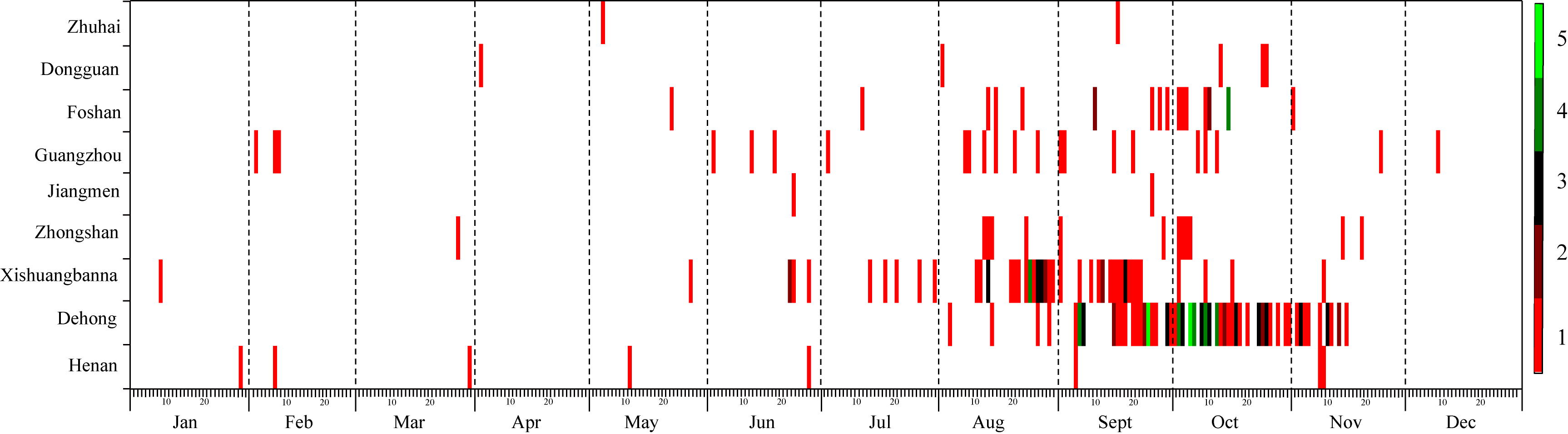

Supplement: S1 Fig — The bar on right side represented the number of dengue cases. (TIF) [file pntd.0005095.s001.tif]

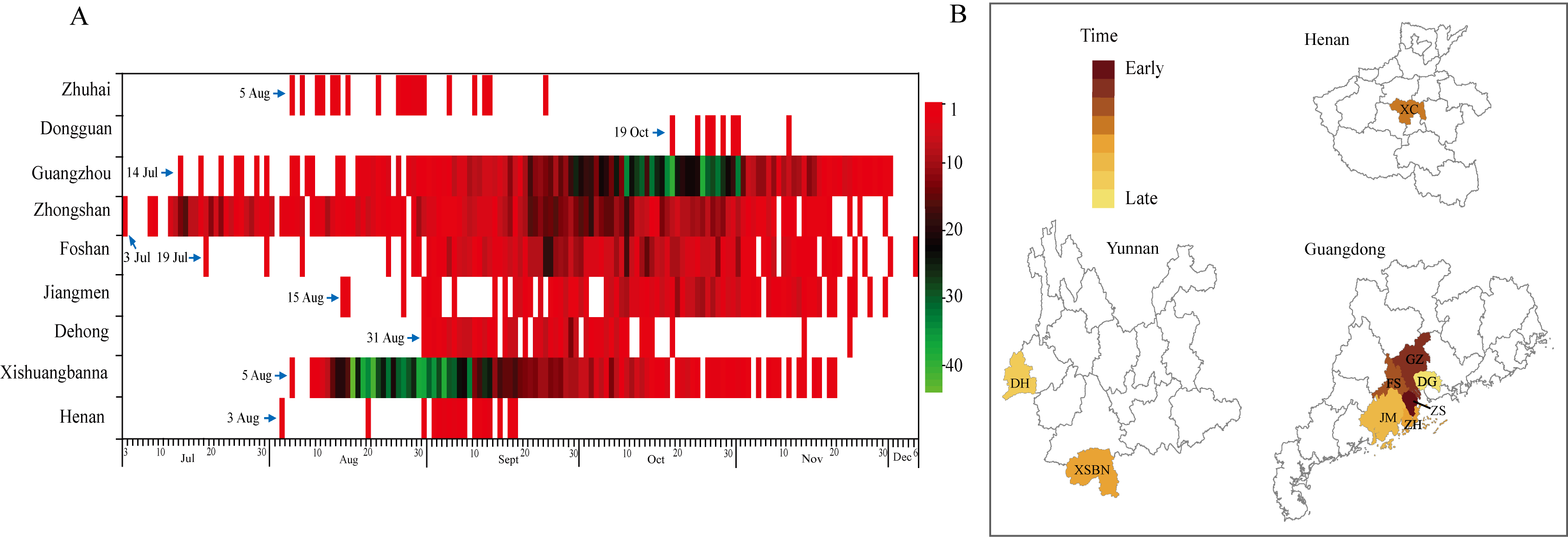

Supplement: S2 Fig — A: Time series of daily local dengue cases. The bar on right side represented the number of dengue cases; B: The plot of relative time of local transmission in every location. (TIF) [file pntd.0005095.s002.tif]

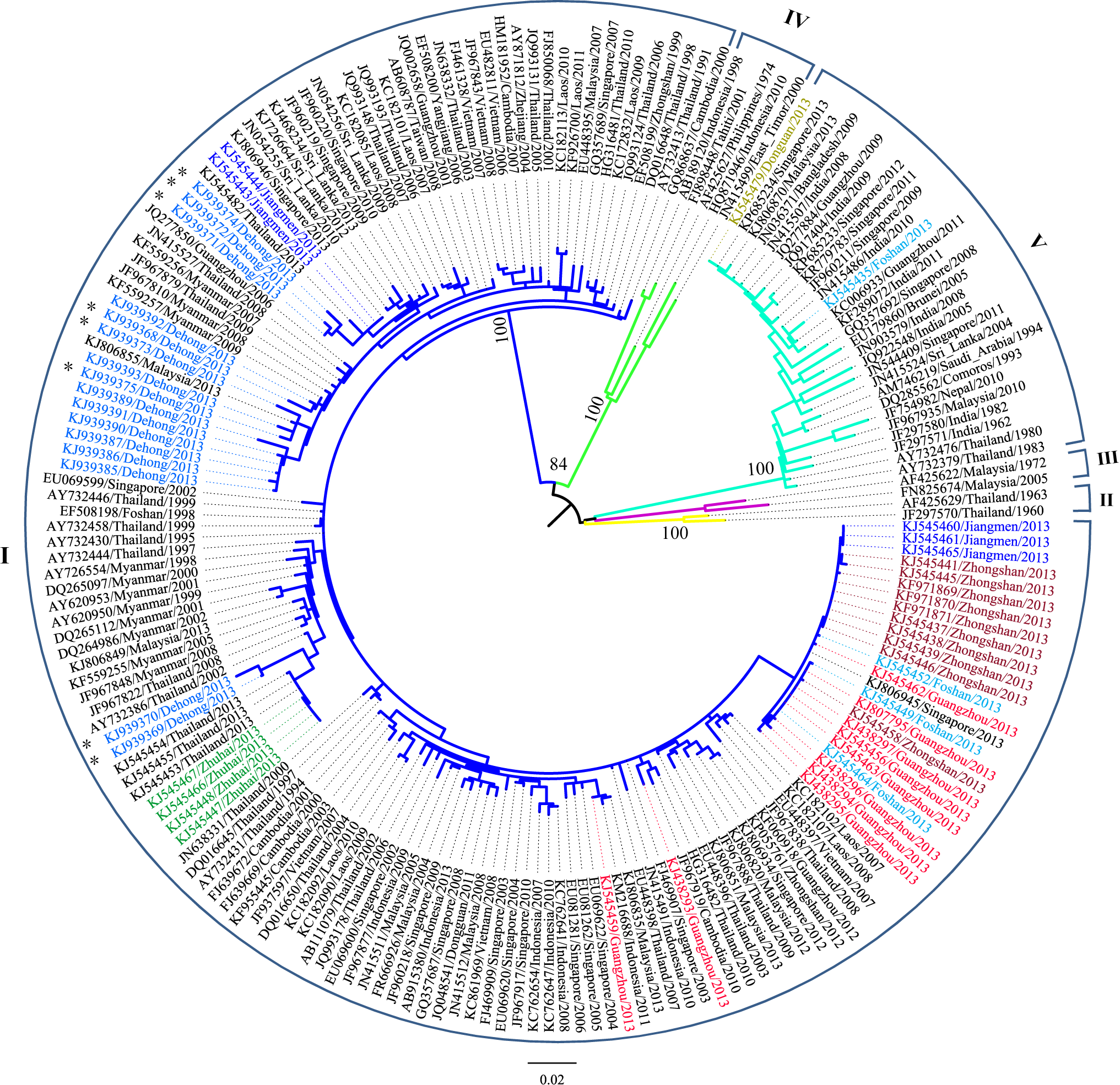

Supplement: S3 Fig — The tree is mid-point rooted. Labs with colors represented strains detected in outbreak areas in China, 2013. Asterisk represented the strains introduced from overseas. (TIF) [file pntd.0005095.s003.tif]

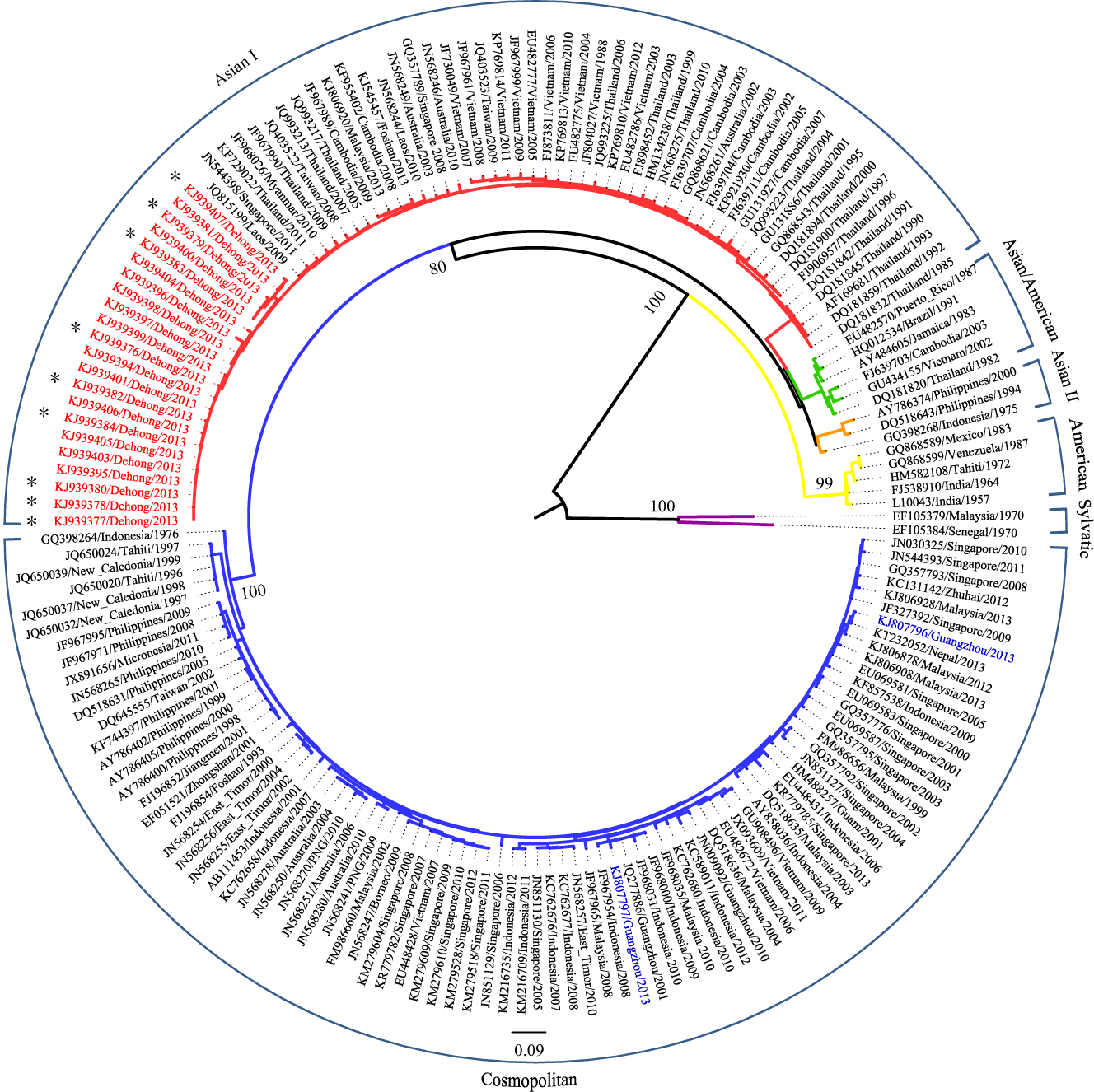

Supplement: S4 Fig — The tree is mid-point rooted. Labs with colors represented strains detected in outbreak areas in China, 2013. Asterisk represented the strains introduced from overseas. (TIF) [file pntd.0005095.s004.tif]

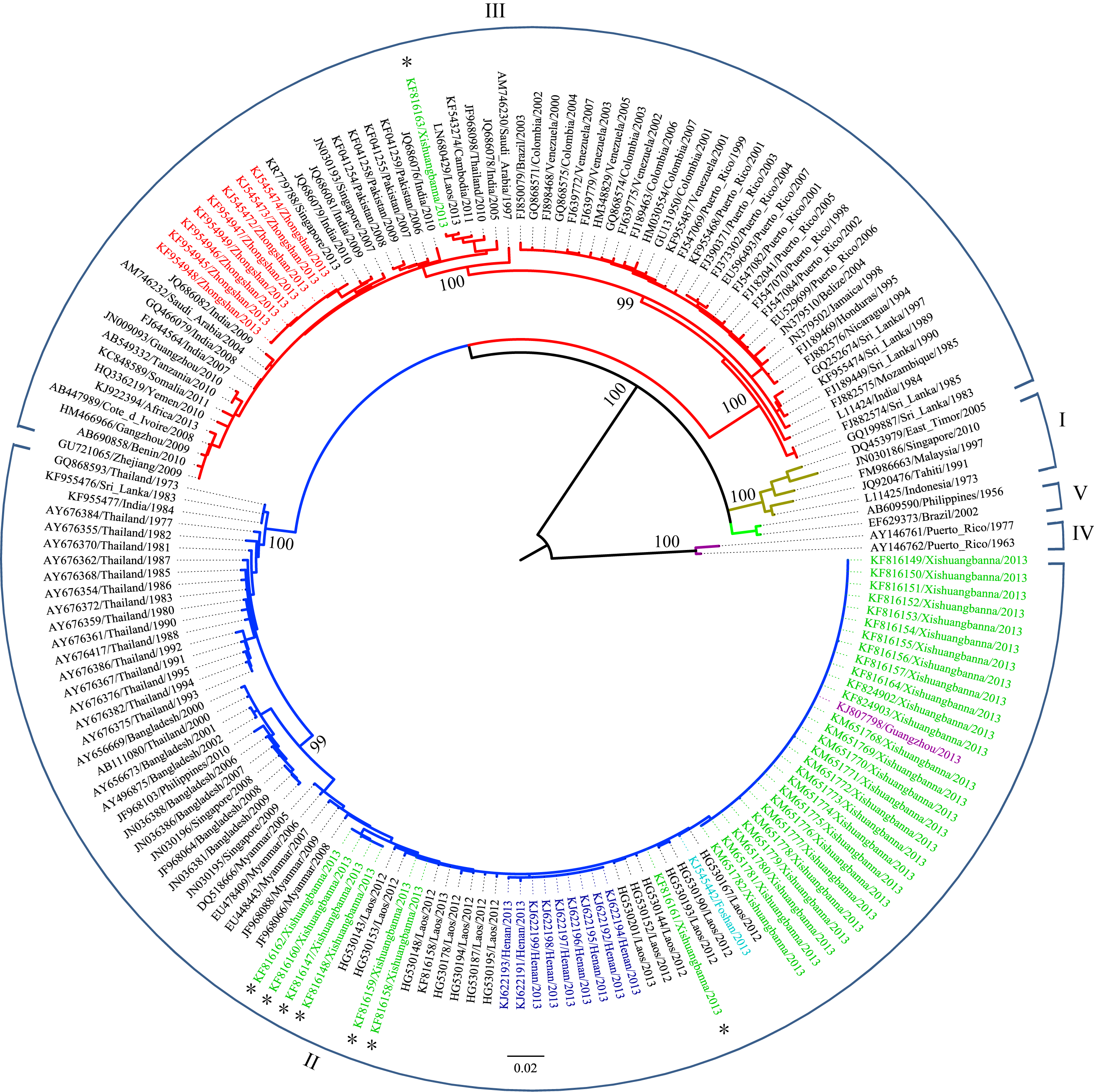

Supplement: S5 Fig — The tree is mid-point rooted. Labs with colors represented strains detected in outbreak areas in China, 2013. Asterisk represented the strains introduced from overseas. (TIF) [file pntd.0005095.s005.tif]
